# Challenges to the implementation of health sector decentralization in Tanzania: experiences from Kongwa district council

**DOI:** 10.3402/gha.v6i0.20983

**Published:** 2013-08-29

**Authors:** Gasto Frumence, Tumaini Nyamhanga, Mughwira Mwangu, Anna-Karin Hurtig

**Affiliations:** 1 Department of Development Studies, School of Public Health and Social Sciences, Muhimbili University of Health and Allied Sciences, Dar es Salaam, Tanzania; 2 Epidemiology and Global Health, Umeå International School of Public Health, Umeå University, Umeå, Sweden

**Keywords:** challenges, decentralization, health services, Tanzania

## Abstract

**Background:**

During the 1990s, the government of Tanzania introduced the decentralization by devolution (D by D) approach involving the transfer of functions, power and authority from the centre to the local government authorities (LGAs) to improve the delivery of public goods and services, including health services.

**Objective:**

This article examines and documents the experiences facing the implementation of decentralization of health services from the perspective of national and district officials.

**Design:**

The study adopted a qualitative approach, and data were collected using semi-structured interviews and were analysed for themes and patterns.

**Results:**

The results showed several benefits of decentralization, including increased autonomy in local resource mobilization and utilization, an enhanced bottom-up planning approach, increased health workers’ accountability and reduction of bureaucratic procedures in decision making. The findings also revealed several challenges which hinder the effective functioning of decentralization. These include inadequate funding, untimely disbursement of funds from the central government, insufficient and unqualified personnel, lack of community participation in planning and political interference.

**Conclusion:**

The article concludes that the central government needs to adhere to the principles that established the local authorities and grant more autonomy to them, offer special incentives to staff working in the rural areas and create the capacity for local key actors to participate effectively in the planning process.

Decentralization, which refers to transfer of power, authority and functions from the central to local authorities (1), has been recognized globally as an important means of improving delivery of public goods and services (2). A World Bank study showed that out of 75 developing and transitional nations having populations greater than 5 million, 63 have initiated reforms aimed at transferring political power to local units of government. The main goal of such reforms was to enhance equity, increase efficiency and ensure more participation and responsiveness of government to citizens (3–11) (see Box 1). However, reviewed literature shows mixed results with regards to the benefits and challenges of decentralization, especially in the health sector. Studies in developing countries (4, 12, 13) reported positive results of decentralization, including increased access to central government resources for people who were previously neglected, particularly those residing in rural regions and local communities, and enhanced participation and increased local administrative capacity to negotiate with central government organs so that they can later allocate more resources for local development activities. In Tanzania, interviewed Council Health Management Team (CHMT) members reported that decentralization had resulted in improvements in training and capacity building for health staff, ability to pay more attention to both supervision and primary health care services and better coordination with donors (14).

In spite of the several reported achievements, there are also reported challenges facing the implementation of health sector decentralization in different countries in the world. The most significant challenge is that in most of the countries, decentralization has not achieved its intended goal of increasing the power of the local authorities because many governments have often implemented de-concentration or delegation forms of decentralization rather than devolution (15). The lack of capacity at local units to implement and manage responsibilities for public services delivery, especially those related to public finances and maintenance of proper accounting procedures, has been reported as one of the factors constraining the implementation of decentralized public services in Uganda and Tanzania (16).

**Box 1. Basic definitions of types of decentralization**

Decentralization is broadly defined as involving the transfer of power, authority, resources, functions and service delivery responsibility from the central government to the lower-level actors and institutions in a political administrative structure (4–8).

Devolution refers to the legal transfer of power to democratically elected local political organs (LGAs) that are supposedly independent of the central government level with respect to a defined set of functions (9, 10).

Deconcentration means that the central government hands over some of its authority to the administrative local offices of the ministry responsible for health. It allows the establishment of local management with a degree of discretion to manage health-related activities without necessarily having constant reference to central government officials (11).

Delegation refers to the transfer of defined managerial and administrative functions and responsibilities to institutions that are outside of the central government organization hierarchical structure. These institutions can be indirectly controlled by the ministry responsible for health (11).

## History of decentralization in Tanzania

In 1962, one year after gaining independence, Tanzania abolished the colonial native authorities (mainly based in urban areas) inherited from the British colony and established democratic local government authorities (LGAs) with the aim of improving the delivery of public goods and services, including health services. During this period, there was no health policy in Tanzania, but both the central and the newly established local authorities implemented a 5-year development plan that emphasized self-reliance and equitable distribution of and access to various social services and resources, including health services (17).

However, 10 years later, the LGAs had failed to achieve the expected results, including eradication of diseases. There were several problems that contributed to this failure, including the expansion of services which did not match with the available financial resources to fund them, a lack of competent personnel, and rampant mismanagement of funds (both funds that were locally generated and those granted by the central government) (18).

The challenges that faced local authorities led to poor social and economic performance, and in 1972 the government of Tanzania adopted a de-concentration type of decentralization whereby power and authority were redistributed to central government agencies at both regional and district levels, which were given responsibility to coordinate both economic and social development activities, including health services (19).

The de-concentration type of decentralization also faced a number of challenges that culminated in its failure in the early 1980s. These challenges included, among others, increased government expenditure; increased bureaucracy in decision making from the central, regional, and district levels to the village level; and a lack of locally generated revenues as the districts were not allowed to collect tax. All these challenges led to the general problem of the slow development of the health sector in Tanzania (20).

Given the deterioration in the delivery of public services, including health services, under the de-concentration system, the government reintroduced local government in 1984 through the Local Government Legislation Act of 1982 (21). However, the reintroduction of the local government adopted a top-down modality, hence it faced some challenges such as central government bureaucracy and the extra power and authority delegated to central government ministries through their regional administrative offices to manage and control social and economic aspects of the local government. As a result, local government again failed to achieve the expected improvements in the delivery of local services, and this suppressed local democracy (22, 23).

In the early 1990s, it became evident that fundamental political, administrative and economic reforms were imperative for the government to be able to improve efficiency and effectiveness in the delivery of public goods and services. Several socio-economic and political reforms were introduced in Tanzania during this period, including establishment of multiparty democracy, liberalization policies (which introduced the free market economy) and introduction of user charges (cost sharing) in the social services, including health (24). Thus, local government and the health sector were among the reforms that were given paramount importance by the 1990s government reforms. This is because of the demand and cry from different local and international stakeholders for an effective decentralization system which would provide an enabling environment for communities at the grassroots level to participate in the decision-making process (25).

Decentralization was one of the most important components of both local government and health sector reforms aimed at transferring key functions, responsibilities, power and resources from the central government to the local authorities, as well as strengthening the capacity of local authorities. In so doing, the government adopted a decentralization by devolution (D by D) strategy, in which LGAs are supposed to be largely autonomous institutions, free to make policy and operational decisions consistent with the country's laws, policies and institutions that have the power to possess both human and financial resources. It was expected that the D by D strategy would yield, among other outputs, the delivery of quality services, including health services, to the people in a participative, effective and transparent way (18). However, since the reintroduction of decentralization in the health sector in the mid-1990s, little has been documented on the challenges facing the implementation of this policy from the perspective of health officials and policy makers at both the national and the local authority levels. Furthermore, reviewed studies have not shown the extent to which decentralization has increased or decreased the choices and discretion of LGAs to make their own decisions regarding council health service plans. The main aim of this article was to assess the benefits and challenges facing the implementation of decentralization of health services in Tanzania using experiences from Kongwa district.

## Decentralization by devolution and decision space approach

In this article, we use the Bossert decision space approach to describe the range of choices for different health-related functions and plans that have been transferred from the centre to the local authorities under decentralized health systems in Tanzania. Bossert proposes the concept of ‘decision space’ as the range of effective choice that is allowed by the central authorities to be utilized by local authorities (26). It can include various functions and activities performed by the local authorities, thereby increasing their degree of choices or discretion to make own decisions (27). The range of local units’ choice or discretion to make decisions over their plans may be defined as ‘narrow’, ‘moderate’ and ‘wide’ (26). As explained here, Tanzania is implementing a devolution form of decentralization; this article is therefore an attempt to describe the benefits and challenges of decentralization by examining the extent to which local authorities have power and authorities to make own choices or discretions over health decisions and plans using a decision space analytical framework (Table 1).

**Table 1:** Map of decision space

	Range of choice		
	
Selected function	Narrow	Moderate	Wide
Finance • Source of revenue • Allocation of expenditure	→ →	→ →	→ →
Planning • Identification of local needs and priorities • Community participation	→ →	→ →	→ →
Service organization • Health facility autonomy	→	→	→
Human resources • Recruitment and contracts • Salaries	→ →	→ →	→ →
Governance rules • Facility boards • Community participation	→ →	→ →	→ →

Source: Modified from the Bossert conceptualization of decision space (26).

## The organization structure for the implementation of the decentralization of health services in Tanzania

There are several organizations involved in the implementation of decentralization of health services in Tanzania. These organizations include both the Ministry of Health and Social Welfare (MoHSW), which is responsible for setting national standards, conducting performance audits and building capacity of facilities for LGAs; and the Prime Minister's Office-Regional Administration and Local Government (PMO-RALG) with the main responsibility of monitoring and coordinating all sectoral activities, in line with the policies and guidelines of the respective ministries. The MoHSW also collaborates with the PMO-RALG and the President's Office Public Service Management to recruit and distribute human resources for health throughout the country. At the regional level, there is the Regional Health Management Team (RHMT) and the social service section under the Regional Secretariat, which perform several activities, including coordinating and advising on the implementation of health policy in the region, monitoring proper management of the health services in the region and building the capacity of LGAs in health service delivery. At the district level, there are various local authority organs, including a Full Council, whose responsibility is to deliberate on and approve district health plans and budgets, and the Council Health Service Board (CHSB), which perform several functions, including discussing and amending district health plans and budgets and identifying and soliciting financial resources for running council health services. The CHMT performs a number of activities such as preparing comprehensive council health plans; ensuring the provision of transport, drugs and medical supplies to health facilities; carrying out supportive supervision to lower level facilities and ensuring the provision of quality health services in the district. The health facility governing boards and committees are important decentralized structures at the grassroots level which perform several functions, including discussing and passing the facility's plans and budget; identifying and soliciting financial resources for running the facility; and advising and recommending on human resources concerning recruitment, training, selection and deployment to relevant authorities (20).

## Methodology

### Study setting

This study was conducted in Kongwa district in Dodoma region situated in the central part of Tanzania. Kongwa is one of the two districts where the health system's research project is being implemented. Kongwa was purposively selected as a typical rural district that has a moderate level of socio-economic development and is fairly accessible in terms of transport and communication networks.

Kongwa district borders Manyara region to the north, Morogoro region to the east, Mpwapwa district to the south and Dodoma rural district to the west.

Administratively, the district has 14 wards, and according to the 2012 Tanzania National Census the population of Kongwa district was 309,977. The health care system in Kongwa district is largely based on public health facilities, including one district hospital, three health centres and 27 dispensaries.

### Study design

A qualitative case study design was employed in this study in order to allow in-depth, comprehensive explorations of the benefits and challenges of the implementation of decentralization of health services in the real-life context (28). As a case study, the district reflects the main ideas on the challenges facing the implementation of decentralization of health services in Tanzania.

### Selection of study participants

Data for this study were collected from key policy makers and planners at the national level and health officials and key local authority officials from the council level. These officials were purposively selected because they were directly involved in making decisions on the implementation of decentralization policy at the national level, and participants from the LGAs were directly involved in the actual implementation of the decentralized health services at the district council level. Specifically, at the national level the study covered a total of seven senior management officials working with the three ministries directly responsible for the implementation of decentralization policy in the health sector: the MoHSW, PMO-RALG and Ministry of Finance (MoF). At the district level, 10 key informants who were directly involved in the implementation and supervision of decentralization of health services at the council levels were interviewed: the district executive officer (DED), the district planning officer (DPLO), the district treasurer (DT), three CHMT members (district medical officer, district health secretary and district health officer), the district AIDS control programme coordinator, two councillors, and the person in charge of the health centre****.

### Data collection techniques

Data collection for this study involved two main sources: interviews with key informants at both the national and district levels and a review of key documents. Interviews with key informants at the district level were carried out between October and December 2011; at the national level, interviews were conducted between January and June 2012. Interviews lasted for between approximately 60 and 90 min and were conducted at the offices of the respondents or in seminar rooms as identified by the interviewees, and both venues provided adequate privacy for the interviews to take place. Two interviewers conducted the interviews, and both of them took notes of the responses. An interview guide was developed and organized into different themes to guide the interviews. The guide comprised questions on knowledge about decentralization and the advantages and challenges of the implementation of decentralization in the health sector at the district level. The interviews were conducted in Kiswahili to reduce language barriers and later translated into English to facilitate joint analysis among all authors.

Several key documents, particularly published articles, books and government policy papers and reports on decentralization policy in general and on the health sector specifically, were purposively selected and reviewed to provide a perspective on the overall decentralization policy as well as the benefits and challenges of its implementation at both the national and local levels.

### Data analysis

A thematic analytical approach was adopted for analysing the data for this article. This approach searches for emerging themes that can describe the phenomenon under investigation (29). It involves the process of carefully reading and re-reading the collected qualitative data (30). In this study, data were analysed manually by the first author in collaboration with other authors by going through the interview and field notes and reviewed documents. The analysis, based on the key emerging themes from the collected data and the reviewed documents, was used to highlight and support key themes that emerged. During data analysis, the authors identified key concepts using inductive coding. These concepts were further analysed to identify their similarities and differences, and they were grouped together to form more precise categories that were later organized into themes. Finally, the results were examined in relation to the Bossert decision space approach (24).

### Ethical issues

The protocol for the study was approved by the Muhimbili University of Health and Allied Sciences. Local research clearance was granted by Dodoma regional administrative officials, Kongwa district officials and ward and village government officials. Oral informed consent was obtained from all the key informants who participated in the study, and they were informed of their right to withdraw from the study at any time. All the interviews were recorded in the notebooks with the permission of each respondent, and the results of the interviews were kept confidential.

## Findings of the study

The analysis of the benefits and challenges of the implementation of decentralization of health services has generated a wide range of issues, which can be classified into two main themes: financial-related benefits and challenges, and managerial and administrative benefits and challenges. Table 2 summarizes the range of choices or discretion given to the LGAs in performing various functions under the D by D approach based on the decision space approach. Supporting quotations from the study participants have been included to illustrate the messages being communicated. The ‘Financial-related benefits and challenges’ section presents findings based on the identified themes.

**Table 2:** Decision space indicators observed in Kongwa District Council

	Range of choice		
	
Selected function	Narrow	Moderate	Wide
Finance			
Source of revenue	CHMT and health facility management teams are entirely dependent on central government allocations.		
Allocation of expenditure		CHMT and health facility management allocate resources to different plans in different cost centres, but the allocation formula between the councils and within the council health expenditure is defined by the central authorities.	
Allocation of expenditure from locally generated income (CHF and cost sharing)			Health facility governing boards and committees have the power to allocate resources to different expenditure items.
Planning			
Identification of local health needs and priorities		CHMT develop and manage plans, but the process is guided by national directives on national health plan priority areas and interference from local politicians.	
Community participation in planning		There is low knowledge among both community members and technical staff on the importance of community participation.	
Service organization			
Health facility autonomy		Participate in planning and deciding on health service delivery, but limited by guidelines stipulated by higher authority	
Human resources			
Recruitment	Permanent staff are recruited and distributed by the central level.		LGAs recruit lower cadre staff only.
Salaries	Defined by national civil service		
Governance rules			
Facility boards	Size and composition of the boards are defined by the Act enacted by the national authority.		
Community participation	The number of service users and representatives of community organizations in the boards is defined by the Act enacted by the national authority.		

Source: Modified from the Bossert conceptualization of decision space mapping (26) to fit in Tanzania's context on decentralization.

## Financial-related benefits and challenges of implementation of decentralization of health services

Study findings revealed one main financial benefit and two challenges of the implementation of decentralization of health services within LGAs. Key informants reported that decentralization has increased autonomy in the mobilization of financial resources from local sources and the possibility of deciding on how to use them for the implementation of health services in the district. One respondent said:

Despite the central government grants, LGAs are now given power to mobilize resources from own sources including community health fund and cost sharing. (District Key Informant, DKI 4)

However, respondents emphasized that the implementation of decentralization of health services is constrained by a lack of adequate financial resources and delays in disbursement of funds from the central government.

### Inadequate funding of local authorities

Respondents from both the national and district levels reported that resources allocated to the councils’ health needs have never been adequate. The government usually provides a ceiling point from which the councils have to budget for their plans. This means that councils are given limitations on how much they should budget for and they must adhere to these ceiling points as one of the criteria for their plans to be approved by the central government. One respondent from the national level commented:

These councils may have more health care needs than those budgeted for, but since the central government has limited resources, then there must be a limit on how much should be allocated to the councils. It cannot make it open for each council to plan and budget without ceiling points. (National Key Informant, NKI 2)

However, the findings from the district council indicate that inadequate financial resources are also caused by a lack of reliable sources of revenue from which the councils could generate their own income. One of the respondents from the district expressed concern about inadequate financial resources:

The central government has allowed councils to collect [their] own revenue from low-yielding tax sources such as property tax, various fees such as meat inspection fee, market fee, and health facility user charges … these sources will never help [the] council to support their social and economic development activities. (DKI 2)

### Delays of disbursement of funds from the central government to LGAs

At the district council level, respondents claimed that the implementation of health service activities is largely dependent on central government funding. The council health department has two main sources of funds from the central government: block grants for personal emolument and other charges, and the basket funds for the operation of service delivery. However, the LGAs complained about excessive delays over funds from the central government, particularly basket funds, as expressed by one of the respondents:

Sometimes we receive the first quota of the funds during the time when we were supposed to get the third quota. This affects very much the implementation of service delivery as some of the planned activities are delayed to start or postponed to the other quarter of the year. (DKI 3)

Respondents from the national level reported that the central government depends on local revenue collections for providing block grants to the LGAs. However, if there are no adequate revenues collected, this will definitely lead to delays in transferring funds to the councils. Regarding delays in disbursing basket funds, the national interviewees said that health basket funds are totally dependent on contributions from development partners. In most cases, donors do not release funds without receiving an annual audited report from the previous year to be able to judge whether the implementation of the activities and the financial management are in line with the procedures and standards that were stipulated in the memorandum of understanding. In this case, one respondent commented:

So if the implementation and audited reports are not submitted on time to the donor community, this will automatically lead to delays in realizing donors’ funds. Ultimately, it will lead to delays in disbursement of basket funds from central government to the LGAs. (NKI 2)

## Managerial-related benefits and challenges of implementation of decentralization of health services

Our key informants mentioned that several managerial benefits resulted from the implementation of decentralization of health services at the council. These included provision of the opportunity for the grassroots community to be involved in the planning process and identify their local priority needs. As one respondent put it,

Decentralization has taken decision-making powers closer to consumers of services. (DKI 6)

Other managerial and administrative benefits of decentralization include enhancement of the accountability of health workers as they are now accountable to the established organs such as CHSB and the Full Council, which are closer to them, instead of the central government. The reduction of bureaucratic red tape in the implementation of health plans, as most of the decisions about health plans are locally made without necessarily involving central government organs as long as the decisions are within approved plans and budget and decentralization, has empowered managers at lower levels to be independent in decision making.

Despite the managerial and administrative benefits that accrued from the implementation of decentralization of health services, the study findings show that there are five managerial-related challenges affecting the implementation of decentralization of health services at the district level. These include a lack of adequate and qualified human resources among LGAs, poor knowledge and skills in planning for both elected and LGA staff, weak supportive supervision, a negative attitude of health workers in involving the local community in planning and managing health service programmes, and political interference in the planning process.

### Limited knowledge and skills among LGA staff and councillors in planning

The study participants claimed that some of the LGA staff were not knowledgeable and skilled enough to develop a comprehensive council health plan. This is exemplified by the following quotation from a CHMT respondent:

Developing a council comprehensive health plan is a technical activity requiring people who are knowledgeable and skilled in planning health-related activities, but our staff have not been well exposed to such type of trainings. (DKI 3)

Similarly, other respondents reported that councillors who constitute the Full Council, which is the supreme decision-making organ of the local authorities, have limited capacity in interpreting the activities planned by the LGA technical staff and making correct decisions. The main reason is that some of the councillors have a low educational level, mainly standard 7, which is the primary education in Tanzania constituting grades 1 to 7, and the ordinary secondary education level, which is the secondary level of education in the country constituting 4 years of schooling from forms 1 to 4.

### Negative attitude of health workers in involving the local community in planning and managing health service programmes

Findings from the district level show that some health workers still think that planning and managing health service activities are the sole responsibilities of health experts, thus representatives of communities cannot take part during the planning process as well as in the management of the health service programmes. One of the respondents said:

Some of the health workers are still obsessed with the old thinking that community members do not know their health needs and priorities because they are not skilled and knowledgeable about health issues. (DKI 1)

### Interference of councillors affects district health plans

Key informants, particularly from the district level, reported that decentralization has enhanced and expanded democracy at the grassroots level by allowing community members to participate in the planning process of their own health needs and priorities. However, they also mentioned that the main challenge of decentralization in the health sector is that the district health planners experience political interference, particularly from the councillors, who sometimes want their respective constituency to be given undue priority. For instance, a councillor may force the construction of a dispensary in his or her constituency to be included in the plan without considering other factors such as availability of staff and other operational costs, which sounds unprofessional, as expressed by one of the key informants:

Sometimes a councillor may ask the planning team to include the construction of a dispensary in his/her constituency without considering that construction of a new dispensary is more than having a building in place. It requires availability of other resources to run it including medical equipment and supplies as well as health workers. (DKI 5)

### Lack of sufficient and technically qualified human resources among LGAs

Almost all respondents from the district level and a few from the national level reported that LGAs do not have enough human resources who are also technically competent to execute and supervise the implementation of the planned activities. A key informant from the district council underscored that the councils greatly affected by this problem are those situated in rural areas which do not attract many potential employees to work there:

Potential employees, especially recently graduated young ones, do not like to work in the rural areas where there are poor working environments, particularly lack of staff houses, no electricity, lack of good office facilities and poor transport. (DKI 1)

### Weak supportive supervision

The study participants, particularly those from the district level, reported that the council health department does not have adequate capacity to carry out supportive supervision at the health facilities and community levels to monitor and control the quality of health service delivery in the whole council. They reported two main contributing factors to this situation: a lack of capacity in terms of transport (inadequate cars, and those that are available are not reliable due to poor maintenance) and inadequate staff to carry out frequent supervision. The following quotation illustrates the point:

Generally the health department has more activities requiring more resources such as transport and human resources than the available resources. … For example, our cars are not enough to carry out supportive supervision to each facility at least once every quarter as planned in our supervision matrix. (DKI 7)

## Discussion of the findings

The discussion of our findings will be guided by the decision space approach, which attempts to describe the range of choice or discretion for different health-related functions and plans that have been transferred from the centre to the local authorities under decentralized health systems in Tanzania (26).

## Financial-related benefits and challenges of implementation of decentralization of health services

### Inadequate funding of local authorities

Tanzania's local government policy paper states clearly that fiscal decentralization in Tanzania is based on a definition of the principles of financial discretionary powers that gives authority to LGAs to levy local taxes, and that the central government has an obligation to supply LGAs with adequate unconditional grants and other forms of grant (21). However, our findings show that LGAs do not have adequate and reliable sources for generating their own resources that can be utilized to support the delivery of health services in the councils. The central government allowed LGAs to generate local revenue from low-yielding tax sources and hence cannot contribute significantly to supporting health service delivery. Our study further revealed that the finance of LGA health services is entirely dependent on central government grants. These grants not only are inadequate to support local health needs and priorities but also are granted to LGAs based on various conditions, including a ceiling point on how much they should budget, and are allocated to different health activities in the council. In most cases, the central government disburses grants late, causing difficulties in the implementation of health activities in the council. A study on local government in Tanzania reported similar findings that the LGAs in Tanzania depend on central government grants for more than 90% of their budgets and that such grants are accompanied by strict and conditional guidelines, directives and instructions (22). A study carried out in Ghana on barriers to implementing health sector administrative decentralization found that the lack of adequate financial resources is one of the main challenges facing decentralization of health services (31). Another study in Uganda also reported that, on the one hand, the central government transferred insufficient funds to the districts to implement the health responsibilities transferred to them, and, on the other hand, the districts do not have adequate and reliable local sources to generate additional resources to support the execution of health activities (32).

## Managerial-related benefits and challenges of implementation of decentralization of health services

### Limited knowledge and skills among the LGA staff and councillors in planning

Our findings show that the limited knowledge and skills in planning among LGA staff and councillors are among the challenges facing the implementation of decentralization in the health sector. Furthermore, health professionals, particularly CHMT members and other health staff, working at the health centres and dispensaries are more active in health service planning, thus dominating the process without much involvement from the communities or their representatives. A study on decentralization and the health care prioritization process in Tanzania (33) also reported that health professionals have a tendency to dominate priority settings for the LGAs, making it difficult for other LGA organs such as CHSB and other stakeholders to make them accountable by asking them questions regarding health service–related operational matters. Another study from Tanzania (25) report that most of the community members or their representatives, particularly in the rural areas, cannot participate fully in the planning process at the grassroots level because they have not been exposed to formal training in planning skills, knowledge and confidence. The findings from this study also noted that health workers were of the view that health plans require people with a professional background in health to be able to competently participate in developing them. However, this perception is contrary to the common thinking that communities should participate in the design and development of the plans that affect their daily lives. Community participation in the design and assessment of primary health care should be considered as a basis of successful implementation of health programmes as well as increasing community ownership of the programmes (34, 35). Thus, this study suggests that by establishing institutional arrangements for the local community to participate in planning without building their capacities by exposing them to planning skills and knowledge, the local units are left with a narrow choice in terms of making decisions and plans on social and economic development activities at their councils.

### Interference of councillors affects district health plans

Our study reveals that decentralization has expanded democracy at the grassroots level by allowing citizens to participate in the planning process to determine their health needs and priorities. However, the planning process at LGAs faces the challenge of political interference, particularly from those councillors who want their constituencies to be given top consideration when prioritizing councils’ health plans. Such political interference distorts the whole process of planning and resource allocation in the councils. The findings from this study are contrary to the common practice of decentralization in most of the developing countries whereby politicians at the national level interfere with and undermine the LGAs’ power, especially any power giving them a wide choice to generate adequate resources, because they feel threatened by the potential power created by new revenue sources (36). These findings suggest that even at the local government level, there is a complex relationship between local politicians and local government technocrats which requires further exploration.

### LGAs’ lack of sufficient and technically qualified human resources

Administrative decentralization in Tanzania is principally supposed to de-link local authority human resources from their respective ministries and directly work under LGAs. This implies that LGAs will have the power to recruit their own personnel who will be accountable to local government, with the expectation that such a system will lead to improved health service delivery (21). The assumption of administrative decentralization is that by giving power to recruit, LGAs will ensure sufficient availability of competent staff according to their human resource requirements. However, the findings from this study present a different experience, indicating that LGAs are suffering from a lack of adequate and technically qualified personnel. This challenge is mainly due to the fact that the role of recruitment, distribution and remuneration of highly skilled human resources is centrally organized, leaving a very narrow choice for LGAs to employ and remunerate lower cadre staff. A study focusing on the decentralization-centralization dilemma and its implications on recruitment and distribution of health workers in remote districts of Tanzania (37) documented similar findings. The centralization of recruitment procedures was done as a way of helping LGAs to secure highly qualified workers in a relatively easy, less complicated and costly manner than in the period before 2006, when LGAs played the role of recruiting and distributing human resources. However, this arrangement denies LGAs the administrative power and authority transferred to them under D by D systems, leaving LGAs with a narrow choice over human resource management functions.

Our study also documents that the lack of power to recruit and determine the salaries of LGA staff results in another challenge, a lack of accountability of some of these staff to the local authorities, which again affects the quality of services delivered.

### Weak supportive supervision

Supportive supervision is recognized by both the central government and LGAs as one of the important activities towards improvement of the quality of health services. In recognition of this, the third Tanzania Health Sector Strategic Plan of 2009 to 2015 states clearly that CHMT supervision of health facilities will focus on coaching based on guidelines and standards, and evidence-based medicine in order to contribute towards improvement of the quality of health services in Tanzania (21). Our findings showed that there is weak supportive supervision from CHMT to lower-level facilities caused by insufficient human resources as well as lack of reliable means of transport. This situation compromises the quality of health service delivery. Similar findings were reported from Malawi, indicating that a high shortage of human resources in the health sector, particularly in the rural areas, affects the quality of health services since there is no frequent supportive supervision (38).

### Strengths and limitations of the study

The strengths of this study emanate from two major factors. The data collection period was prolonged to allow the researchers enough time for reflections between field visits and to perform a preliminary analysis that guided the subsequent data collection, and the final data analysis was facilitated by peer-debriefing sessions involving a multidisciplinary research team.

There are two main limitations of this study. The first one was the unavailability of policy makers and planners at the national level given their busy working schedule. However, in order to ensure that all key informants were interviewed, data collection at the national level took a longer time (more than 4 months). The second limitation was that interviews were not recorded; instead, interviewers took notes of the information collected from the respondents. Despite the fact that the findings of this study were collected from a limited sample of key implementers of decentralization at the district level and decision makers at the national level, we believe that the insights gained from this study are reflective of the general situation in Tanzania. This is due to the fact that the implementation of decentralization in the health sector does not differ between local authorities, implying that information obtained from our key informants may represent challenges facing other LGAs towards the implementation of decentralization of health services in the country. Furthermore, documentary reviews which provide supporting evidence for the analysis of our findings were collected from the national and district levels, and they reflect the status of the implementation of decentralization across the country.

## Conclusion

This article indicates that there are some benefits associated with decentralization of health services. These include increased autonomy in the mobilization of financial resources from local sources, increased powers to decide on how to utilize them, and an enhanced bottom-up approach to health planning by involving the community at the grassroots level to identify their local priority needs. Other benefits include enhancement of the accountability of health workers to the local community bodies such as CHSB and the Full Council, and reduction of bureaucratic procedures by transferring more decision-making power to LGAs from the centre. However, the implementation of decentralization in the health sector is constrained by several challenges at the local level, which require the attention of policy makers and implementers of decentralization policy at the national and district levels, respectively.

First, the central government needs to adhere to the principles guiding the implementation of the decentralization policy as stipulated in the relevant documents and 1982 Act. The government should grant more financial powers to the LGAs to levy local taxes as well as provide them with adequate and unconditional grants to be able to implement social and economic development activities.

Second, the central government, in collaboration with LGAs, needs to design a mechanism for attracting more potential employees to work in the rural areas in order to be able to respond to the citizens’ health needs and improve the quality of care. One of the policy interventions to attract potential employees is to have special incentives for staff to accept work in remote areas.

Third, and finally, this study suggests that more training is required to build the capacity of the established decentralized bodies, particularly CHMT, CHSB and health facility governing boards and committees, to enable them to contribute effectively towards improving the quality of health service delivery.
